# Multiple substance use and blood pressure in women experiencing homelessness

**DOI:** 10.1016/j.abrep.2023.100483

**Published:** 2023-02-13

**Authors:** Leslie W. Suen, Eric Vittinghoff, Alan H.B. Wu, Akshay Ravi, Phillip O. Coffin, Priscilla Hsue, Kara L. Lynch, Dhruv S. Kazi, Elise D. Riley

**Affiliations:** a National Clinician Scholars Program, Philip R. Lee Institute of Health Policy Studies, University of California, San Francisco, San Francisco, CA, United States; b San Francisco Veterans Affairs Medical Center, San Francisco, CA, United States; c Department of Epidemiology and Biostatistics, University of California, San Francisco, San Francisco, CA, United States; d Department of Laboratory Medicine, University of California, San Francisco, San Francisco, CA, United States; e Department of Medicine, University of California, San Francisco, San Francisco, CA, United States; f Department of Medicine, Division of HIV, Infectious Diseases and Global Medicine, School of Medicine, University of California, San Francisco, San Francisco, CA, United States; g San Francisco Department of Public Health, San Francisco, CA, United States; h Division of Cardiology, Chan Zuckerberg San Francisco General Hospital, San Francisco, CA, United States; i Richard A. and Susan F. Smith Center for Outcomes Research, Beth Israel Deaconess Medical Center, Boston, MA, United States; j Harvard Medical School, Boston, MA, United States

**Keywords:** Blood pressure, Cocaine, Hypertension, Substance-related disorders, Women

## Abstract

**Background:**

Substance use increases risk of cardiovascular events, particularly among women with additional risk factors like housing instability. While multiple substance use is common among unstably housed individuals, relationships between multiple substance use and cardiovascular risk factors like blood pressure are not well characterized.

**Methods:**

We conducted a cohort study between 2016 and 2019 to examine associations between multiple substance use and blood pressure in women experiencing homelessness and unstable housing. Participants completed six monthly visits including vital sign assessment, interview, and blood draw to assess toxicology-confirmed substance use (e.g., cocaine, alcohol, opioids) and cardiovascular health. We used linear mixed models to evaluate the outcomes of systolic and diastolic blood pressure (SBP; DBP).

**Results:**

Mean age was 51.6 years; 74 % were women of color. Prevalence of any substance use was 85 %; 63 % of participants used at least two substances at baseline. Adjusting for race, body mass index and cholesterol, cocaine was the only substance significantly associated with SBP (4.71 mmHg higher; 95 % CI 1.68, 7.74) and DBP (2.83 mmHg higher; 95 % CI 0.72, 4.94). Further analysis found no differences in SBP or DBP between those with concurrent use of other stimulants, depressants, or both with cocaine, compared to those who used cocaine only.

**Conclusions:**

Cocaine was the only substance associated with higher SBP and DBP, even after accounting for simultaneous use of other substances. Along with interventions to address cocaine use, stimulant use screening during cardiovascular risk assessment and intensive blood pressure management may improve cardiovascular outcomes among women experiencing housing instability.

## Highlights

• Among women with unstable housing, cardiovascular risk is elevated.
• Cocaine was the only substance associated with elevated blood pressures.
• More intensive hypertension screening and management are needed in this population.

## 1 Introduction

Hypertension is perhaps the most common cardiovascular disease (CVD) and risk factor for cardiovascular events worldwide (Egan et al., 2014). It is independently associated with development of ischemic heart disease, stroke, and heart failure, and it is also one of the most modifiable risk factors for CVD (Egan et al., 2014, Frieden and Berwick, 2011). CVD risk can differ by age, gender, stress, and a variety of sociodemographic and lifestyle factors, including access to health care, prescribed medication use, and the use of controlled substances (Ahmad and Oparil, 2017, Egan et al., 2014, Ghadiani, 2015, Jolaade et al., 2019). Substance use is a known contributor to hypertension, particularly the chronic use of alcohol, tobacco, methamphetamine, and cocaine (Ghadiani, 2015, Kozor et al., 2014, Lv et al., 2016, McFadden et al., 2005), and yet this topic is largely understudied among women.

While substance use is strongly and consistently associated with hypertension, few prior studies have acknowledged polysubstance use or simultaneously estimated independent associations between each substance and elevated blood pressure, particularly in women (Riley et al., 2013, Riley et al., 2017, Riley et al., 2019). This is a large oversight given that (a) over 70 % of people who use substances like cocaine also use additional substances (Liu et al., 2018), and (b) polysubstance use (e.g.*,* the co-use of cocaine and alcohol) can be associated with higher levels of cardiac injury in women who are unstably housed compared to single drug use (Riley et al., 2020). Understanding relationships between polysubstance use and CVD risk factors such as hypertension in women have significant public health implications, particularly as overdose deaths and cardiovascular mortality from polysubstance use are rising (Drug Overdose Deaths, 2019, Rodriguez et al., 2020).

Overdose deaths involving multiple substance use are increasing potentially due to evolving drug use patterns, decreased access to treatment resources, a progressively unsafe drug supply, and more social isolation leading to increased drug use, all of which heighten the importance of effective, evidence-based interventions (Blaney-Koen, 2020). Stimulant toxicity is one of the most common causes of death among women experiencing homelessness and unstable housing (Riley et al., 2013), and cocaine use in particular is strongly associated with small vessel disease, a condition upstream of stroke (Riley et al., 2021). Identifying and systematically tackling contributing mechanisms, sources of risk, and distinct issues for people who use substances is important (McKay & Asmundson, 2020); it could lead to clinically relevant recommendations that target groups at high-risk for cardiovascular morbidity and mortality, especially in women where these conditions are understudied. We therefore conducted a community-based cohort study of women experiencing homelessness and unstable housing to examine associations between the toxicology-confirmed use of multiple controlled substances and blood pressure.

## 2 Methods

### 2.1 Study design and recruitment

Polysubstance Use and Health Outcomes Evaluation (PULSE) is a cohort study of women experiencing homelessness and unstable housing in San Francisco. PULSE data collection was conducted between June 2016 and January 2019 to examine the influence of polydrug use on cardiac dysfunction. PULSE study methods are detailed elsewhere (Riley et al., 2020).

In brief, we enrolled a sample of community-recruited women from homeless shelters, free meal programs, a probability sample of single room occupancy (SRO) hotels and an area sample of street encampments. We also recruited and oversampled women living with HIV to address HIV-specific study aims. Women living with HIV were recruited from the Zuckerberg San Francisco General Hospital HIV clinic (“Ward 86”), a safety net provider of HIV services in San Francisco, and from provider/participant referrals. Inclusion criteria included female sex at birth, aged 18 years or older, and any lifetime history of housing instability, defined as ever having slept in a public place, homeless shelter, or with a series of friends or relatives due to having no other place to sleep (*i.e.,* “couch-surfing”).

Study participants completed six consecutive monthly study visits, with each visit including a confidential interview, vital sign assessment, and lab testing to assess drug use, medication use, cardiac biomarker levels, and HIV status. Participants were reimbursed $40 for each study interview. This study was approved by the Institutional Review Board at the University of California, San Francisco (IRB #14–13868).

### 2.2 Measurements

We present one aspect of the PULSE study here, which investigated the influences of toxicology-confirmed individual and multiple substance use on systolic blood pressure (SBP) and diastolic blood pressure (DBP). We assessed a single blood pressure reading according to a standardized protocol, with patients sitting upright and both feet planted on the floor after a five-minute period of rest and silence. An Omron^TM^ 6-series wrist blood pressure monitor (BP652N) was used for obese patients based on research staff discretion, and an Omron^TM^ 10-series upper arm blood pressure monitor (BP785N) was used for all other patients.

We used Qualitative liquid chromatography-high resolution mass spectrometry (LC-HRMS) to determine the presence of multiple substances in urine samples. The average window of detection is one to three days post ingestion for cocaine, methamphetamines, cannabis, heroin, morphine and other opioids (Verstraete, 2004) and one to five days for alcohol (Helander et al., 2009). We utilized an SCIEX 5600 TripleTOF LC-HRMS system for data acquisition and generation of mass spectra, and we acquired data using HRMS full scan mode with information dependent acquisition of HRMS product ion spectra.

We used untransformed SBP and DBP as continuous primary dependent measures rather than binary measures (e.g., hypertension vs no hypertension) to make more precise clinical interpretations. Primary independent measures included toxicology-confirmed substance use, including cotinine/nicotine (tobacco), alcohol, cannabis, cocaine, methamphetamine, heroin, and additional opioids. Additional independent measures included age; race/ethnicity; individual factors associated with hypertension, including body mass index (BMI), diabetes, prior stroke, prior myocardial infarction (MI), serum levels of high density lipoprotein (HDL) and low density lipoprotein (LDL) cholesterol, C-reactive protein; the presence of cardiovascular medications, including beta blockers, calcium channel blockers, diuretics, and other antihypertensive agents; postmenopausal status; estradiol level; and additional comorbidities including HIV and hepatitis C (HCV) infections. As many study participants were facing socioeconomic and structural stressors, we measured psychological distress and risk for anxiety and depression using the Kessler Psychological Distress Scale (*K*10) as an ordinal variable, with scores of 10–15 interpreted as low level of psychological distress, 16–21 as moderate, 22–29 as high, and 30–50 as very high (Marel et al., 2016).

### 2.3 Statistical analysis

We used linear mixed models to conduct a within-person analysis in which BP at time *t* was predicted by substance use at time *t*. We considered fixed effects of race/ethnicity and age; we considered random effects of all other factors. We estimated associations between repeated measures of toxicology-confirmed substances and SBP and DBP over six study visits. The mixed models included random person-specific intercepts, with the residuals assumed to be independent with constant variance. We first estimated unadjusted associations, and after omitting predictors with p > 0.1 from the unadjusted analysis, we selected a multivariable model using a backwards deletion approach using final retention criterion of p < 0.05. The backwards deletion approach is less likely than alternatives to omit negatively confounded sets of variables (Vittinghoff et al., 2012). Finally, we assessed effect modification by race/ethnicity and concurrent use of other substances. All analyses were done using Stata Version 16.2 (Stat Corp., College Station, TX).

## 3 Results

### 3.1 Participants

We recruited 245 women; the mean age was 51.6 years (standard deviation [SD] 10.8 years) and 74 % were women of color, with 38 % self-identifying as Black/African American, 15 % Latina, 12 % Multiracial, and 9 % other race/ethnicity (Table 1). In terms of cardiovascular profile, 8 % had reported a prior history of myocardial infarction, 12 % a prior history of stroke, and 15 % diabetes. Due to oversampling, 31 % were HIV positive and 32 % had prior or active HCV infections. Participants varied in psychological stress levels, with 14 % scoring low stress, 24 % moderate stress, 36 % high stress, and 26 % very high stress.

**Table 1:** Baseline Characteristics of Study Participants (N = 245).

**Study Characteristic**	**Mean (Standard Deviation) or Proportion (%)**
**Age**	51.6 (10.8)
**Race/Ethnicity**	
White	64 (26 %)
Black/African American	92 (38 %)
Latina^a^	37 (15 %)
Multiracial	29 (12 %)
Other	23 (9 %)

**Comorbidities**	
Post-menopausal b	154 (63 %)
Diabetes c	37 (15 %)
Prior myocardial infarction c	20 (8 %)
Prior stroke c	28 (12 %)
HIV-positive c	77 (31 %)
History of Hepatitis C infection c	78 (32 %)
Chronic Hypertension (Stage 1 or Higher) d	170 (80 %)
Previously or currently taking medications for hypertension c	88 (36 %)

**Psychological Stress**	
Low stress	34 (14 %)
Moderate stress	60 (24 %)
High stress	87 (36 %)
Very high stress	64 (26 %)

**Cardiovascular Measurements**	
Body Mass Index (BMI)	27.9 (23.2–34.0)
LDL cholesterol (mg/dL)	93.0 (77.0–117.0)
HDL cholesterol (mg/dL)	61.0 (47.0–73.0)
C-reactive protein (mg/L)	3.1 (0.9–8.9)
Systolic Blood Pressure	129.0 (115.0–145.0)
Diastolic Blood Pressure	85.0 (77.0–93.5)

**Blood Pressure at Baseline**	
No hypertension	58 (24 %)
Elevated blood pressure (systolic ≥ 120 mm Hg or diastolic ≥ 80 mm Hg)	12 (5 %)
Stage 1 hypertension (systolic ≥ 130 mm Hg or diastolic ≥ 80 mm Hg)	70 (28 %)
Stage 2 hypertension (systolic ≥ 140 mm Hg or diastolic ≥ 90 mm Hg)	102 (41 %)
Hypertensive crisis (systolic ≥ 180 mm Hg or diastolic ≥ 120 mm Hg)	5 (2 %)

**Toxicology Testing at Baseline**	
Cocaine e	129 (53 %)
Cocaethylene	42 (17 %)
Levamisole	83 (34 %)
Methamphetamine	71 (29 %)
Heroin/ Monoacetylmorphine-6	5 (2 %)
Fentanyl/Norfentanyl	6 (2 %)
Additional opioids f	55 (22 %)
Alcohol g	71 (29 %)
Cannabis (THC)	125 (51 %)
Cotinine/Nicotine	169 (69 %)
Benzodiazepine h	21 (9 %)
Naloxone	0 (0 %)
Methadone	53 (22 %)
Buprenorphine/Norbuprenorphine	1 (0.4 %)

**Testing for Multiple Substances**^i^	
Toxicology positive for 0 substances	36 (15 %)
Baseline toxicology positive for 1 substance	56 (23 %)
Baseline toxicology positive for ≥ 2 substances	153 (63 %)

**Cocaine Testing with Other Substances**	
Percent of visits positive for cocaine	60 % (0–100 %)
No cocaine use	116 (47 %)
Cocaine only	2 (0.8 %)
Cocaine with other stimulants only j	14 (6 %)
Cocaine with depressants only k	18 (7 %)
Cocaine with both depressants and stimulants j, k	95 (39 %)

**Other Medications Tested at Baseline**	
Beta blocker l	14 (6 %)
Calcium channel blocker m	10 (4 %)
Other antihypertensive n	11 (5 %)
Statin o	0 (0 %)
Lidocaine	38 (16 %)
Acetaminophen	64 (26 %)
HIV medications	54 (22 %)

^p^ HIV medications including Tenofovir, Emtricitabine, Darunavir, Raltegravir, or Dolutegravir.

a All participants reporting Latina ethnicity regardless of other racial categories mentioned.

b > 1 year since last menstrual period.

c Self-reported.

d Hypertension defined as those who had at least two separate visits with Stage 1 Hypertension (systolic ≥ 130 mm Hg or diastolic ≥ 90 mm Hg).

e Cocaine, Benzoylecgonine, Ecgonine methyl ester or Norcocaine.

f Morphine, Codeine, Hydrocodone, Hydromorphone, Dihydrocodeine, Morphine Glucuronide, Codeine Glucuronide, Promethazine, Oxycodone or Oxymorphone, Tramadol, O-desmethyl-*cis*-tramadol, Meperidine, Normeperidine or Levorphanol.

g As determined by ethyl glucuronide.

h 7-Aminoclonazepam, Clonzepam, Diazepam, Lorazepam, Nordiazepam, Temazepam, Oxazepam, Alprazolam, alpha-hycroxyalprazolam, Flurazepam, 2-Hydroxyethlflurazepam, Desalkylflurazepam, Flunitrazepam, 7-aminoflunitrazepam, *N*-Desmethylflunitrazepam, Midazolam, 7-aminontrazepam or Etizolam.

i Multiple substances including cocaine, methamphetamine, heroin, fentanyl, other additional opiates, alcohol, marijuana, benzodiazepines, naloxone, methadone, and buprenorphine.

j Other stimulants including methamphetamine and nicotine.

k Depressants including alcohol, cannabis, benzodiazepines,^h^ fentanyl, heroin, methadone, buprenorphine and other opiates.

l Metoprolol, Atenolol, Carvedilol, Labetolol.

m Amlodipine, Diltiazem, Verapamil.

n Furosemide, Hydrochlorothiazide, Isosorbide mononitrate, Clonidine, Lisinopril or Losartan.

o Atorvastatin, Pravastatin or Simvastatin.

There was a high prevalence of stage 1 hypertension (indicating elevated risk for cardiovascular outcomes) and stage 2 hypertension (indicating elevated risk for cardiovascular outcomes to the point of needing antihypertensive medication) within the sample. At the baseline visit, 72 % of participants met criteria for at least stage 1 hypertension (SBP > 130 mm Hg or DBP ≥ 80 mm Hg). Among 213 participants with more than one visit, 80 % had at least two study visits meeting criteria for at least stage 1 hypertension, while 55 % had at least two visits meeting criteria for stage 2 chronic hypertension or higher (SBP ≥ 140 mm Hg or DBP ≥ 90 mm Hg). Only 36 % of participants with hypertension reported previously or currently taking any anti-hypertensive medications.

In terms of substance use, at baseline 69 % of women tested positive for nicotine/cotinine, 53 % for cocaine, 29 % for methamphetamine, 29 % for alcohol, 2 % for heroin, 2 % for fentanyl, and 63 % of women were positive for at least two substances. Toxicology profiles were similar across all visits. Of the 129 participants positive for cocaine at baseline, 2 (2 %) tested positive for cocaine only, 14 (11 %) for cocaine with other stimulants like methamphetamine or nicotine, 18 (14 %) for cocaine with depressants like alcohol, cannabis, benzodiazepines, and opioids, and 95 (74 %) for cocaine with both depressants and other stimulants.

### 3.2 Associations between study factors and SBP

Adjusted analysis showed that across all visits, SBP increased significantly as age increased (3.70 mmHg higher per 10 years of age; 95 % CI 1.75, 5.66) (Table 2). It was also significantly higher in Black/African American compared to White participants (10.99 mmHg higher; 95 % CI 5.81, 16.17) and multiracial participants compared to White participants (10.18 mmHg higher; 95 % CI 3.05, 17.32), those with higher HDL (2.60 mmHg higher per SD increase; 95 % CI 1.03, 4.17), those with higher BMI (0.46 mmHg higher per 1-unit increase in BMI; 95 % CI 0.23, 0.69), and in women with positive toxicology for cocaine (4.71 mmHg higher, 95 % CI 1.68, 7.74). SBP was significantly lower in the presence of calcium channel blockers (8.51 mmHg decrease; 95 % CI −13.88, −3.14). Additional substances and risk factors for elevated blood pressure, including prior stroke and prior MI, did not reach levels of significance in this population.

**Table 2:** Longitudinal Associations between Study Factors and Systolic Blood Pressure (SBP).

	**Unadjusted, mmHg**		**Adjusted, mmHg**	
	**Effect (95 % CI)**	**p-value**	**Effect (95 % CI)**	**p-value**
Age, per 10 years	3.63 (1.55, 5.71)	<0.001	3.70 (1.75, 5.66)	<0.001
Race/Ethnicity				
White	Ref		Ref	
Black/African American	13.94 (8.55, 19.33)	<0.001	10.99 (5.81, 16.17)	<0.001
Latina	6.03 (-0.87, 12.93)	0.09	5.89 (-0.64, 12.41)	0.08
Multiracial	10.84 (3.32, 18.36)	0.005	10.18 (3.05, 17.32)	0.005
Other	4.89 (-3.28, 13.05)	0.24	3.94 (-3.71, 11.59)	0.31
Post-menopausal	5.15 (1.23, 9.06)	0.01		
Diabetes	2.29 (-2.69, 7.28)	0.37		
Prior myocardial infarction	−0.13 (-2.55, 2.29)	0.92		
Prior stroke	0.33 (-1.04, 1.70)	0.63		
HIV-positive	−4.34 (-9.15, 0.46)	0.08		
History of Hepatitis C infection	−1.01 (-4.82, 2.80)	0.60		
Body Mass Index (BMI)	0.31 (0.07, 0.54)	0.01	0.46 (0.23, 0.69)	<0.001
LDL cholesterol, per SD (mg/dL)	0.98 (-0.53, 2.49)	0.20		
HDL cholesterol, per SD (mg/dL)	2.79 (1.19, 4.39)	<0.001	2.60 (1.03, 4.17)	0.001
C-reactive protein	−0.41 (-1.10, 0.28)	0.24		
Estradiol	0.00 (-0.02, 0.01)	0.44		
Cocaine	4.48 (1.41, 7.55)	0.004	4.71 (1.68, 7.74)	0.002
Cocaethylene	1.54 (-1.69, 4.78)	0.35		
Levamisole	1.43 (-1.22, 4.08)	0.29		
Methamphetamine	0.30 (-2.74, 3.35)	0.85		
Heroin/Mono-acetylmorphine-6	−0.47 (-6.98, 6.05)	0.89		
Fentanyl/Norfentanyl	1.67 (-4.49, 7.83)	0.60		
Additional opioids	−0.79 (-4.06, 2.47)	0.63		
Alcohol	−2.07 (-4.83, 0.68)	0.14		
Cannabis (THC)	−2.48 (-5.15, 0.20)	0.07	−2.38 (-4.95, 0.20)	0.07
Cotinine/Nicotine	−0.55 (-3.62, 2.52)	0.72		
Benzodiazepine	−4.38 (-8.54, −0.21)	0.04		
Naloxone	−4.36 (-35.51, 26.78)	0.78		
Methadone	−4.69 (-8.75, −0.64)	0.02	−3.76 (-7.66, 0.14)	0.06
Buprenorphine/ Norbuprenorphine	−0.12 (-12.02, 11.78)	0.98		
Beta blockers	−1.79 (-7.03, 3.44)	0.50		
Calcium channel blockers	−7.36 (-12.85, −1.87)	0.009	−8.51 (-13.88, −3.14)	0.002
Other Antihypertensives	−2.33 (-8.06, 3.39)	0.42		
Statin	0.71 (-9.01, 10.43)	0.89		
Lidocaine	0.26 (-2.69, 3.20)	0.87		
Acetaminophen	−1.35 (-3.62, 0.93)	0.25		
Any HIV medication	−1.15 (-4.41, 2.12)	0.49		

### 3.3 Associations between study factors and DBP

Adjusted analysis showed that DBP across all visits was significantly higher in Black/African American participants (4.61 mmHg higher vs Whites; 95 % CI 1.32, 7.89) (Table 3). It was also significantly higher with increasing BMI (0.19 mmHg higher for every 1-unit increase in BMI; 95 % CI 0.04, 0.34), higher LDL cholesterol (1.41 mmHg higher per SD; 95 % CI 0.42, 2.41), higher HDL cholesterol (2.20 mmHg higher per SD; 95 % CI 1.12, 3.27), and in women with positive toxicology for cocaine (2.83 mmHg higher; 95 % CI 0.72, 4.94). DBP was significantly lower in the presence of methadone (4.47 mmHg lower; 95 % CI −7.12, −1.81). Similar to SBP, additional substances and other risk factors associated with elevated blood pressure did not reach levels of significance in this population.

**Table 3:** Longitudinal associations between study factors and diastolic blood pressure.

	**Unadjusted, mmHg**		**Adjusted, mmHg**	
	**Effect (95 % CI)**	**p-value**	**Effect (95 % CI)**	**p-value**
Age, per 10 years	1.10 (-0.23, 2.43)	0.10		
Race/Ethnicity				
White	Ref		Ref	
Black/African American	6.89 (3.47, 10.31)	<0.001	4.61 (1.32, 7.89)	0.006
Latina	0.99 (-3.40, 5.38)	0.66	0.63 (-3.51, 4.78)	0.76
Multiracial	5.14 (0.35, 9.93)	0.04	3.04 (-1.51, 7.59)	0.19
Other	−0.16 (-5.36, 5.03)	0.95	−1.34 (-6.20, 3.53)	0.59
Post-menopausal	1.91 (-0.67, 4.49)	0.15		
Diabetes	0.31 (-3.03, 3.65)	0.86		
Prior myocardial infarction	−0.61 (-2.33, 1.11)	0.49		
Prior stroke	0.22 (-0.76, 1.20)	0.66		
HIV-positive	−2.69 (-5.69, 0.31)	0.08		
History of Hepatitis C infection	−1.73 (-4.25, 0.80)	0.18		
Body Mass Index (BMI)	0.14 (-0.01, 0.30)	0.07	0.19 (0.04, 0.34)	0.02
LDL cholesterol, per SD (mg/dL)	1.58 (0.56, 2.60)	0.003	1.41 (0.42, 2.41)	0.005
HDL cholesterol, per SD (mg/dL)	2.41 (1.34, 3.49)	<0.001	2.20 (1.12, 3.27)	<0.001
C-reactive protein	−0.20 (-0.69, 0.29)	0.43		
Estradiol	0.00 (-0.01, 0.01)	0.83		
Cocaine	2.28 (0.19, 4.38)	0.03	2.83 (0.72, 4.94)	0.009
Cocaethylene	−0.20 (-2.48, 2.08)	0.86		
Levamisole	1.06 (-0.79, 2.91)	0.26		
Methamphetamine	1.27 (-0.84, 3.38)	0.24		
Heroin/Mono-acetylmorphine-6	0.16 (-4.48, 4.80)	0.95		
Fentanyl/Norfentanyl	2.21 (-2.16, 6.58)	0.32		
Additional opioids	−0.77 (-3.11, 1.56)	0.52		
Alcohol	0.90 (-1.02, 2.83)	0.36		
Cannabis (THC)	−1.40 (-3.26, 0.46)	0.14		
Cotinine/Nicotine	−0.12 (-2.24, 2.00)	0.91		
Benzodiazepine	−1.82 (4.47, 1.10)	0.22		
Naloxone	2.65 (-19.61, 24.91)	0.82		
Methadone	−4.94 (-7.66, −2.23)	<0.001	−4.47 (-7.12, −1.81)	<0.001
Buprenorphine/ Norbuprenorphine	−0.86 (-9.29, 7.56)	0.84		
Beta blockers	−1.27 (-4.89, 2.35)	0.49		
Calcium channel blocker	−3.20 (-7.08, 0.67)	0.11		
Other Antihypertensive	−1.74 (-5.74, 2.25)	0.39		
Statin	−1.24 (-8.24, 5.75)	0.73		
Lidocaine	−0.86 (-2.96, 1.24)	0.42		
Acetaminophen	−0.08 (-1.71, 1.55)	0.92		
Any HIV medication	−0.61 (-2.86, 1.65)	0.60		

### 3.4 Effect modification and differences by stress

In our effect modification by race/ethnicity analysis, there was no evidence that the race/ethnicity modified the association of cocaine with SBP (p = 0.21) or DBP (p = 0.63). Similarly, we did not find significant effect modification of cocaine effects on blood pressure by concurrent use of other stimulants, depressants, or both (SBP: p = 0.21; DBP: p = 0.39) compared to those who used cocaine only (Appendix A). Finally, stress as measured by *K*10 scale was not associated with significant higher SBP or DBP in this population (Appendix B).

## 4 Conclusions

In this prospective study of women experiencing homelessness and unstable housing, over half had chronic stage 2 hypertension. Cocaine was the only substance significantly associated with higher SBP and DBP, even after adjusting for age, race, BMI, cholesterol, other substance use, stress, and other factors associated with elevated blood pressure. Thus, results indicate that the independent association with cocaine alone stood out as being especially strong. Additionally, known risk factors, including alcohol, tobacco, prior stroke, prior MI, and HIV, were not significantly associated with changes in blood pressure. These results suggest that, while multiple substance use is a known risk factor for a variety of outcomes, a stronger clinical and research focus on cocaine use in low-income women may have a larger population-level impact on blood pressure control.

The inclusion of very high-risk individuals led to two surprising findings. First, while over 55 % of participants had chronic hypertension signaling the need for antihypertension medication, only 14 % had toxicology-confirmed presence of calcium channel blockers, beta blockers, or other antihypertensive medications. Participants may have been prescribed antihypertensive medications but not been taking them recently or taking antihypertensive medications not included in our chosen panel. However, the number of participants with hypertension who self-reported taking any antihypertensive medications was also low at 36 %. This low prevalence of preventive cardiovascular treatment represents an opportunity for more intensively addressing hypertension control in this population. The second surprising finding was no significant association between psychological stress and hypertension, potentially due to varying patterns of stress in this unique population that may not be adequately captured by the *K*10 (Furukawa et al., 2003).

While prior studies have shown individual substances associated with higher blood pressure (Egan et al., 2014, Kozor et al., 2014, Lv et al., 2016, McFadden et al., 2005), our study is the first to concurrently examine how the use of multiple substances relates to systolic and diastolic blood pressure. Our results confirm that cocaine stands out as the substance having the strongest association with blood pressure, and that cocaine effects are not modified by other drugs. This finding is highlighted by the relevance of cardiovascular morbidity and mortality in drug-related deaths, where a large portion of sudden cardiac arrests are attributed to the use of multiple substance use and drug overdoses, and stimulants like cocaine are increasingly playing a role (Rodriguez et al., 2020). It also extends prior studies reporting that cocaine use stands out from other substances as being strongly associated with cardiac injury, small vessel disease, and fatal overdose in women experiencing homelessness and unstable housing (Riley et al., 2013, 2020, 2021). Taken together, the existing evidence suggests that incorporating routine assessment of stimulant use—particularly cocaine use—as a core risk factor for cardiovascular and cerebrovascular events during health care encounters, and intensive hypertension management when appropriate, may reduce morbidity and mortality in this population (Riley et al., 2022).

Cocaine use has made a resurgence in the United States in recent years, and cocaine-related overdose deaths are on the rise (Hedegaard et al., 2018, John and Wu, 2017). From 2014 to 2016, the number of overdose deaths involving cocaine almost doubled from 5,892 to 11,316 (Hedegaard et al., 2018). Cocaine is known for its deleterious effects on the cardiovascular system, and cocaine use has disproportionally increased in populations already at risk for cardiovascular morbidity, including Black/African American communities, cisgender women, those older than 50, and those reporting simultaneous cocaine use with alcohol (John & Wu, 2017). This is especially noteworthy for women experiencing homelessness and unstable housing, given that cocaine-related cardiovascular events are the most common cause of death in this population (Riley et al., 2013). Further, recent widespread reports of increased substance use associated with COVID-19-related stress and anxiety are only expected to persist (Banducci and Weiss, 2020, McKay and Asmundson, 2020, Spagnolo et al., 2020).

The high rates of cardiovascular mortality in this population, combined with the emphasis on cocaine reported here, suggest several potential implications. First, given that traditional CVD risk stratification tools do not account for substances outside of tobacco or alcohol, our results suggest that screening and evaluation for cocaine use and hypertension are especially important among populations that include high proportions of women experiencing homelessness. Ongoing research and health interventions including risk evaluation and health care delivery can be further tailored to meet the needs of people who use drugs, who often face barriers to outpatient care engagement. For example, contingency management is an evidence-based behavioral treatment shown to be effective in reducing stimulant use (Rawson et al., 2006). However, it has not been historically offered in primary care or cardiology clinic settings; offering contingency management in chronic cardiovascular disease care represents an opportunity for further exploration (DesJardin et al., 2021).

Second, providers in multiple disciplines, including primary care clinicians, specialists, nurses, frontline clinical staff, social work, among others, may consider counseling women using multiple substances on the especially noteworthy effects of cocaine use relative to other substances, and when appropriate, prioritize cocaine reduction and cessation as a first step to reducing CVD risk. While abstinence from all substances is an important goal, complete cessation may not be sustainable or feasible for all individuals, and studies have reported low rates of drug cessation among low-income populations mostly due to systemic factors such as higher rates of housing instability, medical and mental health comorbidities, prior trauma, etc. (Rapp et al., 2006). A harm reduction approach may be considered to counsel women using multiple substances, especially those with high cardiovascular risk, to prioritize cocaine reduction, rather than cessation, to minimize cardiovascular morbidity. Third, given the lack of evidence-based effective pharmacological therapies for cocaine cessation, prioritizing intensive blood pressure management in this population may reduce risk for future cardiovascular events.

This study has several limitations, including potential unmeasured confounding due to the observational nature of the study. However, randomized trials of substance use effects are largely unethical, and the current study conducted more thorough adjustment than is often undertaken. The sample size was also relatively small. Nevertheless, this cohort using community-based sampling represents one of the largest samples of stimulant-using women to study drug use and CVD. Finally, the effect of cocaine on SBP and DBP was clinically small, though even small effects result in clinically meaningful outcomes over time with chronic use (Kim & Park, 2019). Future studies to estimate the long-term effects of cocaine use on cardiovascular outcomes such as chronic hypertension would illuminate these initial findings which mainly quantify short-term effects. Strengths of the study include the use of toxicology-confirmed testing of substances, prescription drugs, and use of multiple measures for each participant over time.

The independent associations between cocaine use and higher systolic and diastolic blood pressure found here highlight the importance of cocaine in the context of cardiovascular risks from multiple substance use. Reducing the use of cocaine, patient counseling, aggressive blood pressure management, and provider assessment of substance use beyond tobacco and alcohol may improve risk stratification and improve the health of low-income women.

Disclosure Statement: All authors contributed significantly to study design, data collection, data analysis, or manuscript preparation, and all authors approve of the manuscript. Dr. Hsue reports honoraria received from Gilead and Merck related to work outside of the submitted manuscript, and no other conflicts declared. The views expressed in this article are those of the authors and do not necessarily reflect the position or policy of the Department of Veterans Affairs or any of its academic affiliates.

Author Contributions: LWS and EDR conceived and designed the study. EDR collected the data. AHBW, POC, PH, KLL, and DSK advised on methods of data collection. EV performed the analysis. LWS and EDR the paper. All authors contributed to manuscript revision, read and approved the submitted version.

Funding: This study was supported by the National Institute on Drug Abuse (R01 DA037012 and K24 DA039780) and the National Heart, Lung and Blood Institute (R38 HL143581). The funding agency had no role in study design, data collection, analysis, the decision to publish, or the preparation of the manuscript.

## Declaration of Competing Interest

The authors declare that they have no known competing financial interests or personal relationships that could have appeared to influence the work reported in this paper.

## Data Availability

Data will be made available on request.
